# Epidemiological trends of type 2 diabetes in Middle East and North Africa, 1990–2023: global burden of disease data analysis

**DOI:** 10.3389/ijph.2026.1609710

**Published:** 2026-07-28

**Authors:** Awad Alshahrani, Raed Aldahash, Badr F. Al-Khateeb, Noof Alwatban, Maryam Alhabas, Seema Nasser, Lubna Alnaim, Ashraf El-Metwally

**Affiliations:** 1 King Abdullah International Medical Research Center, Riyadh, Saudi Arabia; 2 College of Medicine, King Saud Bin Abdulaziz University for Health Sciences, Riyadh, Saudi Arabia; 3 Ministry of the National Guard-Health Affairs, Riyadh, Saudi Arabia; 4 College of Public Health and Health Informatics, King Saud Bin Abdulaziz University for Health Science, Riyadh, Saudi Arabia; 5 Department of Nursing, College of Nursing, King Saud Bin Abdulaziz University for Health Sciences, Riyadh, Saudi Arabia; 6 College of Applied Medical Sciences, King Saud Bin Abdulaziz University for Health Sciences, Al Ahsa, Saudi Arabia

**Keywords:** DALYs, global burden of disease, Middle East and North Africa, mortality, prevalence

## Abstract

**Objective:**

This study systematically evaluating temporal trends of type 2 diabetes (T2DM) prevalence, mortality, and DALY estimates from countries in MENA (Middle East and North Africa) region.

**Methods:**

The involved a secondary analysis of Global Burden of Disease (GBD) 2023 data regarding age-standardized prevalence, mortality, and DALY rates are calculated for men and women by country and sub-region using 1990 and 2023.

**Results:**

Overall, prevalence of T2DM increased from 5.6% in 1990 to 11.5% in 2023. Diabetes-related deaths, peaked between 2010 and 2015, declined to approximately 30 per 100,000 by 2023, disability burdens (DALY rates) rose, among men. Regional variations include rapid increases in Turkey, high rates in Levant, upward trends in North Africa (notably Egypt and Tunisia), and highest DALYs in Saudi Arabia and Bahrain (2,600 to 3,400 per 100,000). Iran and Yemen reported low but increasing disability burdens.

**Conclusion:**

T2DM has been consistently increasing over last 30 years in MENA region and has presented itself as a more debilitating and lethal chronic disease over this period. There is a wide range of variation between countries within the MENA region.

## Introduction

Type 2 Diabetes Mellitus (T2DM) is a major global health issue in the 21st century, significantly affecting populations across clinical, social, and economic dimensions [1, 2]. It leads to various complications that result in early mortality and long-term disability, primarily impacting the cardiovascular, renal, nervous, and ophthalmic systems [3, 4]. The rising prevalence of T2DM is linked to demographic shifts, urbanization, dietary changes, reduced physical activity, and environmental factors, highlighting the resilience and preparedness of health systems to manage its economic burden [5, 6].

The Middle East and North Africa (MENA) region is undergoing rapid demographic and economic changes, leading to an increase in T2DM [7, 8]. Contributing factors include high obesity rates, sedentary lifestyles, western dietary patterns, and air pollution, all of which elevate cardiometabolic risks [9–11]. Evidence indicates that morbidity, mortality, and disability rates related to T2DM are rising in various MENA countries, with many cases linked to modifiable risk factors [12–14]. Numerous studies on T2DM in the MENA region reveal critical gaps. Most focus on singular aspects like prevalence or incidence without integrating mortality and disability metrics [15]. Additionally, much of the data is outdated or collected over limited time frames, hindering long-term trend analysis [16]. Cross-country comparisons are often inadequate, obscuring different national trajectories and intervention needs. Lastly, few studies analyze age-standardized trends across prevalence, mortality, and disability-adjusted years, despite the recognized importance of such metrics for assessing progress and equity [17].

This study highlights critical gaps in data on the prevalence, mortality, and disability-adjusted life years (DALYs) associated with T2DM in the MENA region. It emphasizes the urgency to address these gaps, particularly as health systems face increasing challenges from chronic diseases and an aging population. Previous analyses primarily utilized data up to 2019, making the period from 2020 to 2023 vital for understanding the impact of the pandemic on chronic disease management [16]. The period from 2020 to 2023 represents a critical epidemiological window, as the disruptions in healthcare services, shifts in population health behaviors, and the long-term metabolic consequences of the global pandemic era have likely altered the trajectory of chronic disease management. By evaluating trends in T2DM from 1990 to 2023, this comprehensive assessment aims to inform evidence-based policies and guide disease prevention strategies, ensuring that emerging vulnerabilities receive necessary interventions.

## Methods

### Design and data source

This study utilizes a systematic analysis framework, defined as the consistent and standardized application of the Global Burden of Disease (GBD) 2023 dataset to track longitudinal epidemiological trends. By employing the GBD’s standardized methodology—which uses rigorous statistical modeling to synthesize data from multiple sources—we ensure that our estimates of prevalence, mortality, and DALYs are both internally consistent and internationally comparable across the MENA region. This framework enables objective identification of health trends from 1990 to 2023, minimizing biases from country-specific reporting. The GBD Study, coordinated by the Institute for Health Metrics and Evaluation at the University of Washington, offers consistent estimates of incidence, prevalence, mortality, and population health for diverse diseases and injuries across demographics and time periods, using data from the GBD 2023 Study through the Global Health Data Exchange Results Tool (https://vizhub.healthdata.org/gbd-results/) [18].

The GBD Study utilizes various data sources, including population surveys, hospital discharge data, administrative claims databases, and systematic literature reviews to standardize the measurement of T2DM disease burdens by year, gender, and geography. It groups MENA countries into sub-regional clusters for better visualization and comparison of epidemiological trends while focusing on the region’s overall burden. Comprehensive methodological details are available in published literature [19–21].

### Primary outcomes and target region

We compiled the data of all available data on the T2DM burden by country and region from 1990 to 2023. Our primary outcomes were prevalence, DALY and mortality due to type 2 DM along with age-standardized rates (ASR) and 95% uncertainty intervals (UI) (Supplementary Table 1). Uncertainty intervals were derived using 1,000 posterior samples drawn during each of the estimation processes/simulations. The 2.5 and 97.5 percentiles represent the distributions we created based on 1,000 posterior samples. For the purposes of data visualization, the countries of the MENA region have been grouped into logical sub-regional clusters (e.g., Turkey, the Levant, North Africa, and the Gulf and adjacent West Asian countries). This sub-regional approach allows for a clearer comparison of heterogeneous epidemiological trajectories while maintaining our overarching focus on the comprehensive MENA burden.

### Case definition and disease classification

To characterize T2DM, we used the GBD health outcome definition. The GBD Study has created a methodology that applies algorithms to data sources with different case definitions for T2DM to remove inconsistencies between countries and to guarantee comparability between the methods used to provide us with each country’s data regarding T2DM. Data were extracted from the GBD 2023 Study via the Global Health Data Exchange Results Tool. The cause of interest was defined as Diabetes mellitus type 2 (GBD Cause Code: B.8.1.2). Our query parameters were configured to extract Age-standardized rates (per 100,000 population) for Prevalence, Mortality, and DALYs, spanning the years 1990–2023 across both sexes. Hence there is a consistent and comparable dataset for the comparison of T2DM-related cases across countries and overtime.

### Data extraction specifications

Data were extracted from the GBD 2023 Results Tool using the following systematic query parameters:

- Cause: Type 2 diabetes mellitus (Cause Code: B.8.1.2)
- Metric: Percent (%), Rate (per 100,000)
- Measure: Prevalence, Mortality, Disability-Adjusted Life Years (DALYs)
- Age Group: Age-standardized (Global Standard Population)
- Sex: Male, Female, Both
- Location: MENA Region (including country-specific codes for [Jordan, Lebanon, Palestine, and Syria, Saudi Arabia, UAE, Qatar, Kuwait, Bahrain, and Oman, Iran, Iraq, Yemen, and Afghanistan])
- Time Period: 1990–2023

A summary of the custom query used for this study can be accessed via the following GBD Results Tool persistent link: https://vizhub.healthdata.org/gbd-compare/.

### Prevalence, DALYs, and mortality

The estimates of prevalence, DALYs, as measured by disability adjusted life years for T2DM, and mortality owing to T2DM were obtained directly from the GBD 2023 Study. In this analysis, the burden of T2DM was not determined from the literature because the GBD has provided a novel statistical method for calculating prevalence, mortality, and DALY estimates through Bayesian meta-regression model. Additionally, the use of multi-period and geographically proximate data has been employed in acquiring T2DM prevalence estimates with less instability when primary data is limited. Prevalence estimates are summarized by age, sex, geography, and constructed by region within the world.

To account for the differences in age distribution of populations across countries, all population prevalence, mortality, and DALY rates were adjusted to allow for comparison using direct standardization using the World Standard Population developed by GBD. The reporting of DALY and prevalence rates include DALY rates are presented as the number of DALYs per 100,000 people; prevalence is reported as a percentage of total numbers of prevalent cases, and mortality is reported as number of deaths per 100,000 population. The absolute changes, percentage changes and annual average growth rates for temporal trends are the differences over 33 years from 1990 to 2023.

### Model uncertainty and handling of missing data

The GBD 2023 Study utilizes a unique methodological approach for the analysis of missing data. Burden estimates of T2DM were calculated based on multilevel regression models that incorporated temporal and spatial autocorrelation. Imputation in data-limited settings was informed by covariate data from several sources (including: the Socio-demographic Index (SDI), age structure of the countries, indicators of healthcare access, and country-specific epidemiological patterns) [22, 23]. Uncertainty from the input data variability, model specification, and disability weight estimation across all modelling stages has been propagated through posterior sampling [18, 22, 23]. The final uncertainty intervals provide a complete range of potential values around the point estimates.

### Differences by sex, time and geography in burden estimates

The total burden estimates the stratification of burden estimates by sex so that differences in T2DM burden can be evaluated by gender, using T2DM -related DALYs and prevalence data. The entire duration of the study from 1990 to 2023 is used to demonstrate trends over a long period and more recently, where they have been stable. Estimates for each country were assessed separately in the MENA region. An analysis of comparative data across countries helps to identify constant high burden countries, rapidly growing burden countries, relatively stable burden countries, and countries experiencing a decline in burden over time.

### Statistically and visually supported analysis techniques

The current study used GBD-based burden estimates by employing a descriptive epidemiological design methodology. To visualize long-term DALY, mortality, and prevalence trends, time-series plots are created for the countries included in MENA region. For males and females, the overall pattern of burden over time for both groups is also illustrated. All analysis followed the GBD analytical protocols to verify that they will be replicable and comparable with prior studies demonstrating global burden of disease.

## Results

### Trends in age-standardized prevalence of type 2 diabetes mellitus in the Middle East and North Africa region, 1990–2023

**FIGURE 1:**
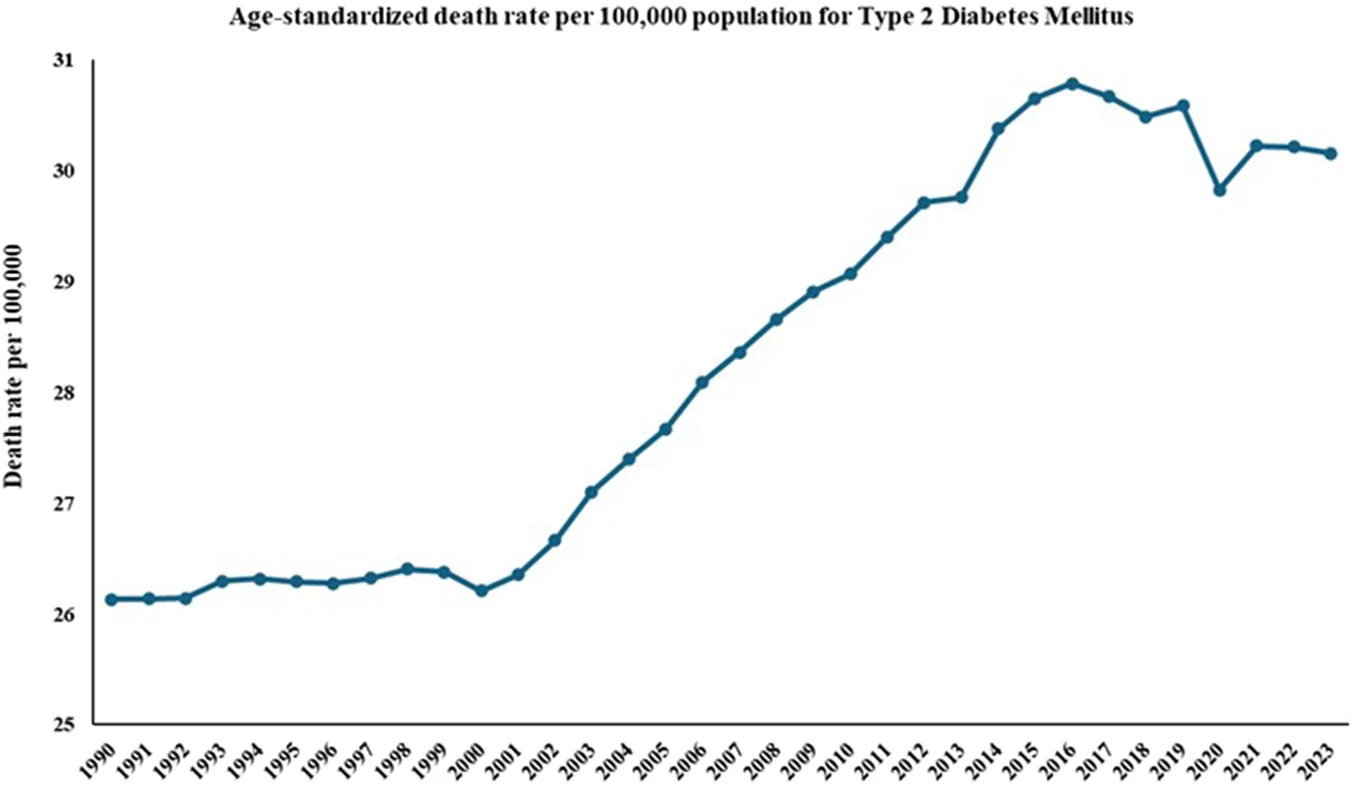
Trends in age-standardized prevalence of type 2 diabetes mellitus in the MENA region, 1990–2023.

Figure 1 illustrates that from 1990–2023, type two diabetes mellitus became an increasingly common health issue in the MENA region. Over the course of three decades, the age-standardized prevalence of type 2 diabetes has more than doubled. In 1990, the prevalence of type 2 diabetes was 5.57% (95% confidence interval: 5.08%–6.05%). Year by year, the prevalence rate increased, reaching 9.29% in 2014, then 10.53% in 2020, and finally 11.51% in 2023 (95% confidence interval: 10.64%–12.45%).

### Age-standardized mortality rate due to type 2 diabetes mellitus in the Middle East and North Africa region, 1990–2023

**FIGURE 2:**
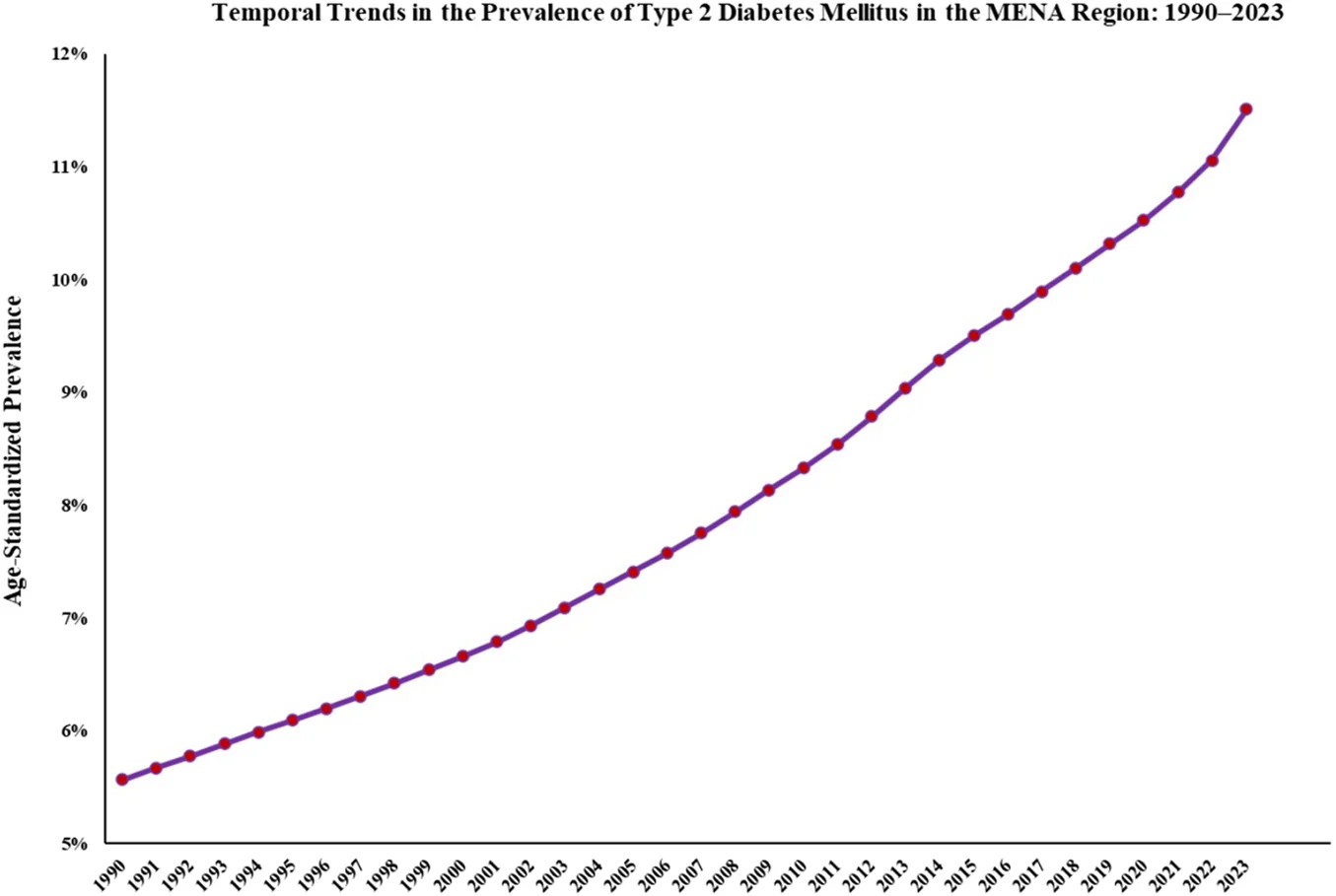
Trends in age-standardized mortality rates (per 100,000 population) for type 2 diabetes mellitus in the MENA region, 1990–2023.

Figure 2 reveals that mortality rates (age-adjusted) attributable to type 2 diabetes have also increased over the same period in the MENA region. In 1990, the age-standardized rate for diabetes-related mortality in the MENA region was 26.13 deaths per 100,000 (95% C.I. 16.89–36.93). By 2014, that figure had climbed to 30.38 deaths per 100,000 (95% C.I. 25.31–36.21). The highest occurrence of diabetes-related mortality within the MENA occurred in 2016 with a rate of 30.79 deaths per 100,000 (95% C.I. 25.87–37.12). Since then, there have been slight decreases and stabilization; by 2023, there were approximately 30.16 deaths per 100,000 (95% C.I. 24.36–36.34) related to type 2 diabetes within the MENA region.

### Sex specific trends in age-standardized DALYs per 100,000 population due to type 2 diabetes mellitus in the MENA region, 1990–2023

**FIGURE 3:**
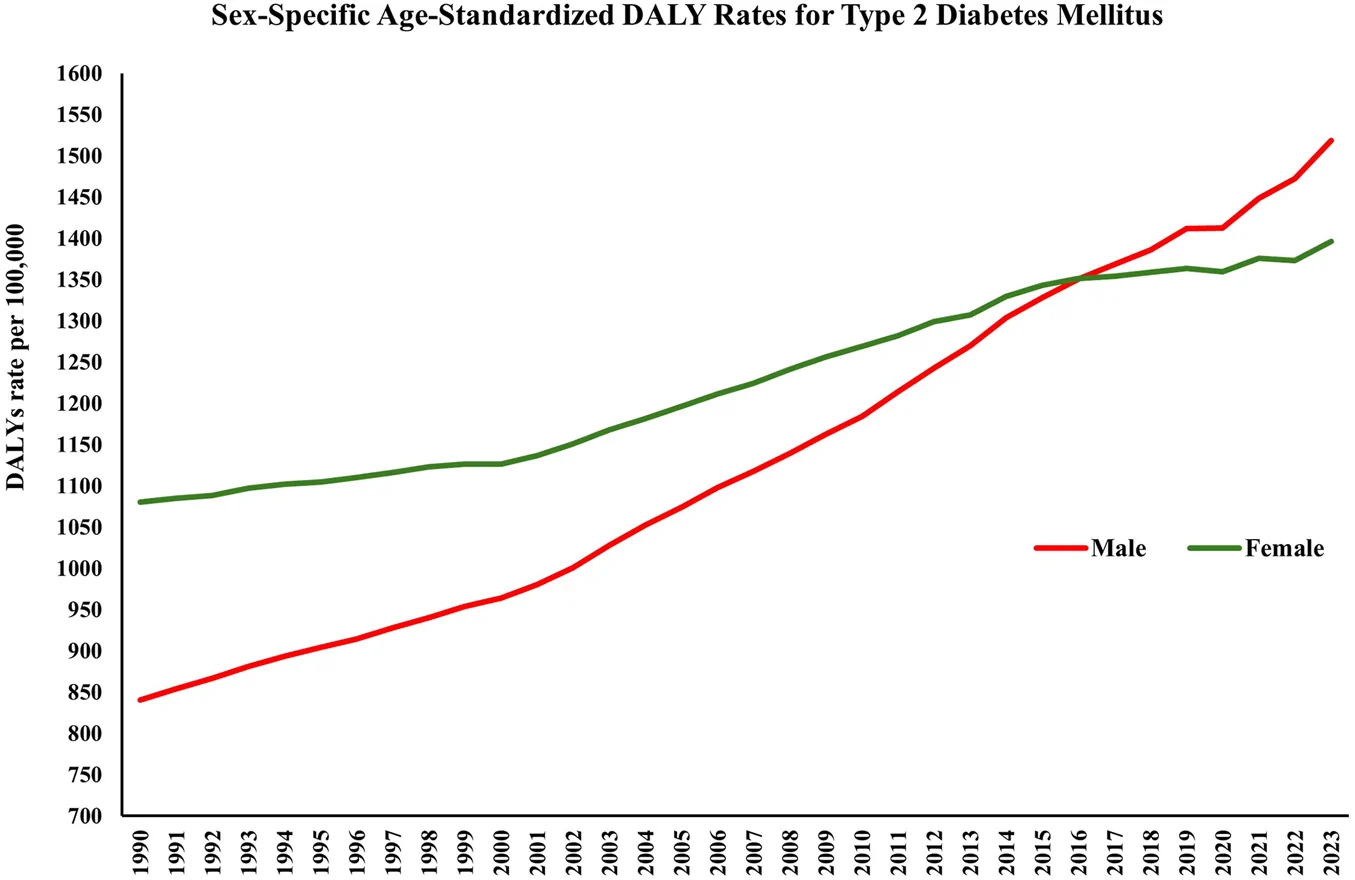
Sex-specific trends in the age-standardized burden of type 2 diabetes mellitus in the MENA region, 1990–2023: a disability-adjusted life year (DALY)-Based analysis.

Figure 3 shows a significant increase in the number of age-adjusted disability-adjusted life-years (DALY) per 100,000 population from type 2 diabetes Mellitus between 1990 and 2023 for both men and women in North Africa and the Middle East region; however, the increase was much more substantial for men than women. Males with type 2 diabetes had a DALY of 840.53 (95% UI: 625.03–1,049.52) in 1990 and an increase of 1,518.81 (95% UI: 1,207.31–1,858.03) in 2023, representing an increase of nearly 81%. For female diabetics, the increase was much less, increasing from 1,080.63 (95% UI: 787.86–1,434.10) in 1990 to 1,396.25 (95% UI: 1,066.15–1,752.78) in 2023, about a 29% increase overall. The trends also indicate that, over the years, the sex gap in diabetes-related DALY has narrowed.

### Age-standardized DALY rates for type 2 diabetes mellitus in Turkey, the Levant, and North Africa

**FIGURE 4:**
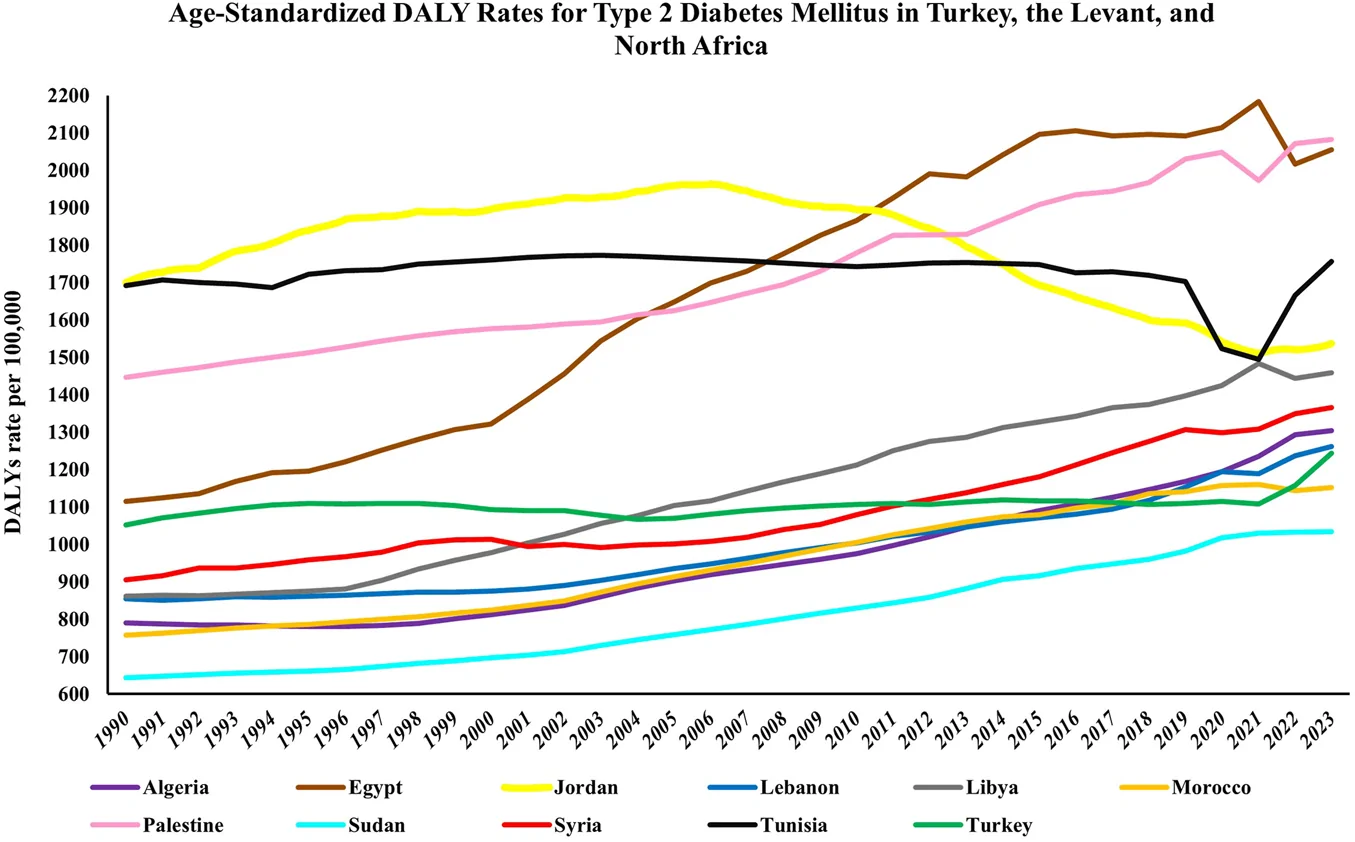
Country-specific trends in age-standardized disability-adjusted life year (DALY) rates (per 100,000 population) for type 2 diabetes mellitus in Turkey, the Levant, and North Africa, 1990–2023.

As shown in Figure 4, across 1990–2023, DALY rates for type 2 diabetes mellitus grown markedly in Turkey, the Levant, and North Africa, nevertheless with apparent regional patterns. Turkey displayed a long period of relative stability from 1990 to 2015 (1,052.54 per 100,000 to about 1,120 DALYs per 100,000), followed by a sharp acceleration after 2019, reaching its maximum rate in 2023 (1,243.07 per 100,000), representative a recent and marked worsening of diabetes burden.

The Levant region was consistently ranked as the area that exhibited the best overall rate increase for this measure during the entire time period with the highest quantified number of incidents of type 2 diabetes being in Palestine (1800–2000 per 100,000; DALY rates show a steady increase until around 2020, followed by a sharp decline thereafter) and in Jordan (1900 per 100,000; climax was reached at around the same time), both of which decreased relatively steadily throughout the rest of the 2000 to 2020 decade until arriving at what was described by the 2023 data as elevated but stabilized rate(s) (Jordan: 1,537.42 per 100,000; Palestine: 2083.03 per 100,000). Lebanon and Syria also exhibited consistent and steady increases in type 2 diabetes rates throughout the entire period as these countries continue to bear increasing burdens of chronic disease and system stresses as their populations age. The North African region also exhibits consistent upward trends across the individual countries within this area; DALY rates have been increased overall 50%, 80% in North Africa since 1990.

Egypt and Tunisia experienced large absolute increases, while Algeria, Morocco, Libya, and particularly Sudan experienced a similar but somewhat less dramatic increase than their North African counterparts. Taken as a whole, these results clearly demonstrate that all three of these regions exhibit a continuously expanding burden for type 2 diabetes, with the Levant region continuing to carry the highest burden of the chronic disease burden over an extended time period, North Africa represents an area undergoing a continued transition of epidemiologic/conditions of health, and currently Turkey appears to be approaching the beginning of an accelerated phase of increasing burden.

### Age-standardized DALY rates for type 2 diabetes mellitus in Gulf and adjacent West Asian countries

**FIGURE 5:**
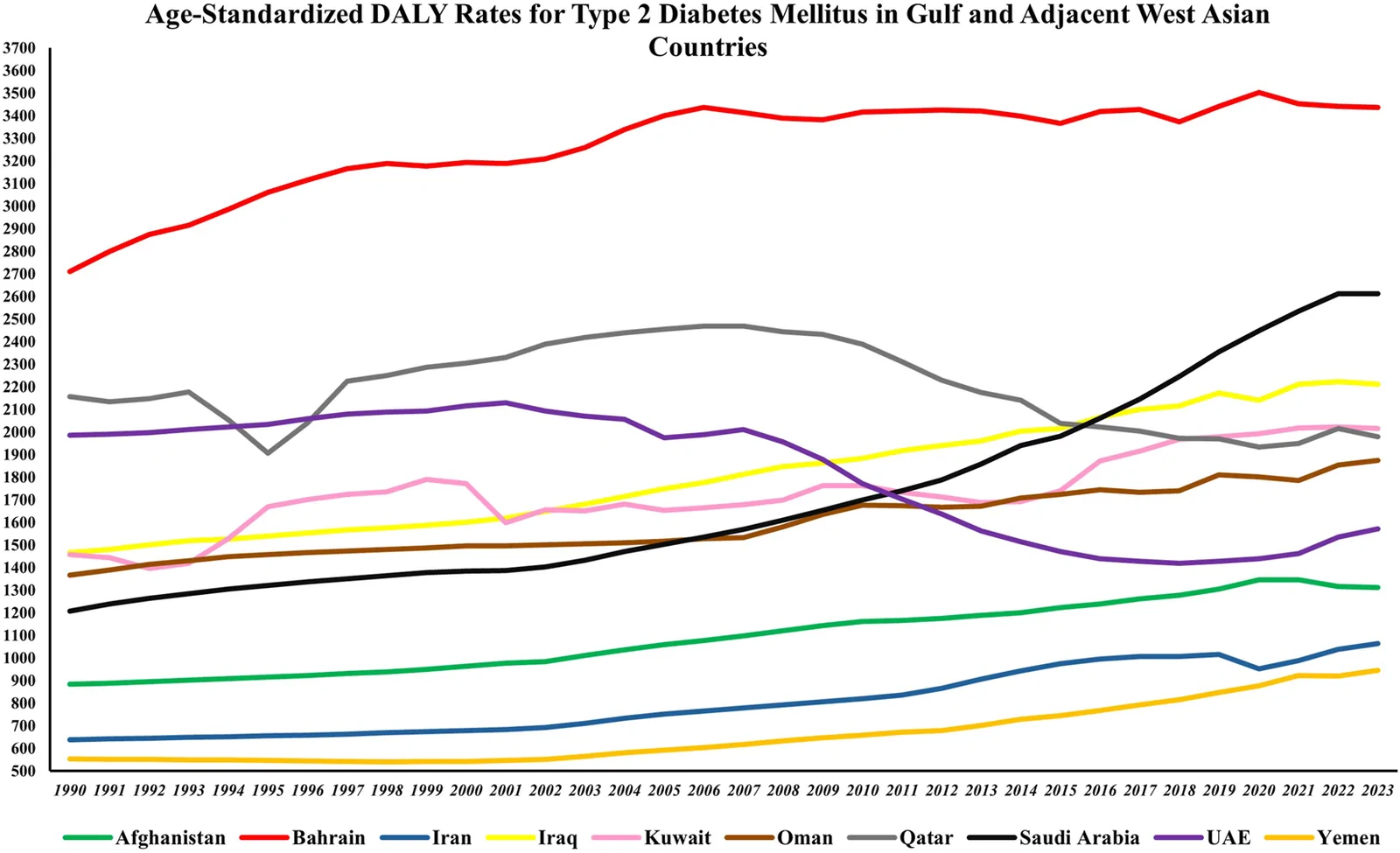
Country-specific trends in age-standardized disability-adjusted life year (DALY) rates for type 2 diabetes mellitus in the Gulf and adjacent West Asian countries, 1990–2023.

As shown in Figure 5, Within West Asia and the Gulf Region, Saudi Arabia, Qatar, Bahrain, and the UAE had consistently the highest age-standardized DALY rates of T2DM, with Saudi Arabia, and Bahrain showing the highest rates (2,613 and 3436 DALY/100,000 people, respectively) by 2023, which is more than a 100% increase over 1990 levels (1,207 and 2,710.5, respectively). In fact, Saudi Arabia has by far the largest burden of T2DM within the Gulf Region and West Asia. The pre-1990 data for Saudi Arabia is limited but based on available information; it can be expected that Saudi Arabia had an increase in DALY rates (i.e., >1207 DALY per 100,000 people), as reported after 1995; Bahrain has maintained consistently high DALY rates throughout this time period with no clear evidence that this will decrease in the future. Also of note is that just like Saudi Arabia, there has been an overall upward trend in the burden of T2DM in both Qatar and the UAE; Qatar peaked in approximately 2006–2008 at >2400 DALY per 100,000 people, before declining to 1979.46 DALY per 100,000 people by 2023; whereas the UAE has steadily increased from 1985 to 1570 DALY/100,000 people, with acceleration in the increase beginning after 2010.

## Discussion

This study analyzes trends in T2DM in the MENA region from 1990 to 2023, highlighting significant increases in prevalence, mortality, and DALYs with significant differences between male and female populations. It indicates T2DM has transformed from a nascent public health challenge to a significant chronic disease impacting health across the MENA region.

The severity of the T2DM burden observed in the MENA region must be contextualized against global trends. While global data from the GBD 2019 study indicates a significant rise in T2DM burden, with global age-standardized DALY rates increasing from 628.3 per 100,000 in 1990 to 801.5 per 100,000 in 2019 [1, 24], Our findings indicate that the MENA region consistently sustains T2DM rates that exceed global benchmarks, reflecting a more severe public health crisis than in many other regions. The region’s disease burden among adolescents and young adults is escalating more rapidly particularly in low-to-middle sociodemographic index (SDI) countries [2], highlighting the need for tailored, aggressive mitigation strategies rather than standard global responses.

An important finding is that the age-standardized prevalence of T2DM increased during this period and now exceeds the prevalence observed at baseline (1990). This increase, consistent with prior studies [16], is likely attributed to the combination of ongoing demographic transition, rapid urbanization, economic development and changes in lifestyle behaviors producing accelerants to cardiometabolic disease risk [15, 25]. Increases in energy-dense diets, decreased physical activity, an increase in obesity, and increased exposure of populations to environmental and psychosocial stressors are all accompanying increased incidence rates of T2DM [26, 27]. The increased survival and the increased rates of case detection following an increase in access to diabetes screening and healthcare may have contributed to the increased incidence of diabetes observed during this period, as improved access to healthcare has resulted in improved diagnostic yield [28, 29]. However, the continued rise in rates indicates that prevention measures have not yet outpaced the number of people at risk for developing T2DM in this region.

It is essential to note that the sex-based differences in T2DM burden, particularly the higher DALY rates observed among males, may be partly attributed to the unique demographic structure of several MENA countries. In the Gulf states, the large influx of male expatriate workers creates a gender-skewed demographic profile [30]. Because many of these individuals are in age groups that may have higher occupational or lifestyle-related metabolic risks, this demographic composition may disproportionately influence the male-to-female ratio in our findings. Therefore, our observation of a higher male burden should be interpreted with caution, acknowledging that it likely reflects a combination of biological factors, health behaviors, and the specific socioeconomic and demographic landscape of the region.

The attributable mortality from T2DM has also increased over time and hit a peak in the mid-2010s before leveling off at a high point. Although there continues to be a sizeable number of diabetes-related deaths, advances made in clinical management, diagnosis, and access to medications have likely prevented additional upward movement but there has been little-to-no improvement recently [29, 31]. While our study design does not permit causal attribution, the observed plateau in diabetes-related mortality warrants further investigation. It is hypothesized that advancements in clinical management, such as the increased availability of SGLT2 inhibitors and GLP-1 receptor agonists, may have contributed to this trend. However, given our ecological study design, this observation should be viewed as a potential contributor rather than a direct causative factor, and future longitudinal clinical studies are needed to confirm these associations [32, 33]. Nonetheless, the ongoing high rates of diabetes-related deaths indicate ongoing issues in providing timely diagnosis, uninterrupted care and adequate management of diabetes as a long-term condition. Many MENA countries have fragmented healthcare systems, a lack of healthcare providers and inconsistent access to medications which are impeding the outcomes for people with diabetes [34].

The significant increase in diabetes-related DALYs illustrates the growing burden of non-fatal diabetes and highlights the critical role diabetes has in producing years lived with disability. DALYs have increased for both males and females, consistent with existing literature [35]. However, the current study revealed a much greater rate for males, indicating changes in the way diabetes-related risk is being distributed over time and potentially differences based on sex in health behaviors, work-related exposures, patterns of seeking care, and other chronic illnesses associated with diabetes. In addition, sub-regional heterogeneity in DALYs highlighted the unique epidemiological and health system dynamics that exist across the globe. Turkey had a long period of low or no change in DALYs until 2019, where a rapid increase occurred, potentially due to rapid lifestyle changes, an aging population, and disruptions from the COVID-19 pandemic to chronic disease management [36, 37]. In the Levant, diabetes DALYs remained among the highest globally, with notable patterns observed in Palestine and Jordan, where there were early peaks followed by declines due to improvements in public health programming, an expansion of primary care services, or shifts in population structure [38–40]. On the other hand, Lebanon and Syria maintain a high level of DALYs related to diabetes as a result of compounded factors from unstable economies, disruption of healthcare services due to conflict, and barriers in providing continuous care for chronic diseases [10, 41, 42].

Our findings show a temporal correlation between regional DALY trends and shifts in health system performance. While we observe a decline in certain areas, we cannot definitively attribute this to specific public health programs using the current dataset. These trends may be influenced by multiple factors, including changes in diagnostic practices, healthcare infrastructure, and shifts in population demographics. We acknowledge that our analysis cannot isolate these individual health system variables, and these interpretations should be considered as potential contextual explanations for the observed data.

North Africa generally showed a consistent increase in T2DM burden across the region, and the greatest increases were seen in Egypt and Tunisia. As with the Levant, the increases in burden can be attributed in part to accelerated nutritional transitions, urbanization, and increases in the prevalence of obesity [43–45]. In Sudan, Morocco, Algeria, and Libya, these same trends exist, but at a slower rate due to variable levels of transition in epidemiological phases and capacity of the healthcare system. Taken together, North Africa demonstrates a region going through metabolic transition, with a risk of continued increases in diabetes burden as effective prevention policies are not implemented on a large scale. In West Asia and the Gulf, T2DM is a significant problem. Saudi Arabia and Bahrain have high DALYs for diabetes, which have more than doubled. Rapid economic development, an abundance of food, a sedentary lifestyle and rising obesity rates are thought to be some of the factors contributing to these trends [27, 31, 46]. The healthcare systems in these countries have increased their capacity for diagnosis but cannot prevent the onset of T2DM.

In Qatar and the UAE, there has also been a significant increase in the number of DALYs for diabetes, although Qatar has experienced a decline after a rise due to possible changes in targeted health intervention practices or the demographic composition of the country. Yemen and Iran continue to have low levels of DALY related to diabetes, but they are also expected to have a growing burden of diabetes. The differences in dietary habits, age distribution, socioeconomic status and the incidence of under-diagnosis may explain the low levels of diabetes in these countries [10, 47]. Many factors may contribute to the increase in T2DM in this region including but not limited to urbanization, reliance on motorized transportation, and changes in the food supply chain due to ultra-processed food are all structural contributing factors [47, 48].

In addition, the observed regional patterns, such as the plateauing of mortality rates in the Levant compared to the sustained, rapid growth in the Gulf region, likely reflect distinct stages of the epidemiological transition. In the Gulf states, the rapid economic development and transition to sedentary, Westernized lifestyles have likely outpaced public health preventative interventions. Conversely, the patterns observed in the Levant and North Africa may reflect a complex intersection of aging populations, constrained health system resources, and ongoing humanitarian or economic instabilities. While our ecological design cannot isolate these specific variables, these patterns clearly delineate the diverse public health ‘fronts’ within the MENA region, underscoring the urgent need for tailored national strategies that address these distinct, localized metabolic risks.

It is also necessary to interpret our findings for the 2020–2023 period within the context of the COVID-19 pandemic. The pandemic introduced substantial systemic shocks that likely influenced the observed trends in T2DM burden. First, the diversion of healthcare resources to manage the acute COVID-19 crisis may have disrupted routine diabetes care, including screening, diagnostic services, and long-term monitoring, potentially leading to an under-representation or delayed documentation of new cases [49]. Second, there is evidence of a ‘syndemic’ interaction, wherein individuals with T2DM faced an increased risk of severe morbidity and mortality when infected with SARS-CoV-2. Consequently, the observed mortality and DALY rates during this period may reflect not only the progression of diabetes itself but also these collateral pandemic-related impacts. While our study does not isolate these pandemic effects, they are important contextual factors that suggest the recent trajectory of T2DM burden is inextricably linked to the broader challenges of health system resilience during the crisis.

### Strengths and limitations

This study presents several strengths: it utilizes 33 years of standardized burden estimates for analyzing temporal trends, employs age-standardized measures for comparability across diverse demographics, integrates prevalence, mortality, and DALYs for a multidimensional view of diabetes burden, and offers sub-regional data disaggregation that highlights geographic disparities and informs policy priorities.

There are several limitations in the study’s estimates, which are based on modeled data that may be influenced by under-reporting, varying surveillance quality, and differing diagnostic criteria for diabetes across countries. Additionally, data quality issues in low-resource and conflict areas could introduce uncertainty in trend estimates. The estimation of Disability-Adjusted Life Years (DALYs) depends heavily on the assumptions used in the methodology, potentially misrepresenting the burden of diabetes. The reliance on ecological data restricts causal inferences regarding the factors influencing diabetes mortality and DALYs, making the interpretations exploratory and cautionary. Furthermore, while the Global Burden of Disease (GBD) framework allows for standardized comparisons, it may overlook localized data nuances, suggesting that findings should complement rather than replace data from national health surveillance systems.

### Conclusion and policy implications

T2DM is a rapidly expanding and increasingly disabling public health issue within the MENA region. Over the last three decades, the increasing prevalence, continued high rates of mortality, and rapidly increasing burden of DALYs strongly indicate an urgent need for the establishment of coordinated prevention, early detection, and sustainable chronic disease management systems. Given the significant contribution of T2DM to regional mortality and long-term morbidity, it must be prioritized as a major driver of chronic disease burden. If strong policy measures are not taken to address this growing public health crisis, there will likely be escalating pressures on the healthcare system and profound socioeconomic consequences across the MENA region.

The study highlights the importance of comprehensive strategies to combat the increasing prevalence of Type 2 Diabetes Mellitus (T2DM) through initiatives that target obesity reduction, healthy eating, physical activity, and social determinants of metabolic risk. It emphasizes the need for improved early screening and diagnosis programs, access to medications, and long-term follow-up to mitigate complications. The sustained high Disability-Adjusted Life Years (DALY) in Saudi Arabia indicates significant gaps in secondary prevention and diabetes management, calling for a shift to integrated chronic care. The study advocates culturally tailored community health interventions and the incorporation of diabetes prevention into broader public health policies and planning. Lastly, it underscores the need for enhanced health information systems to assess the diabetes burden and evaluate prevention program effectiveness, providing a foundation for future studies on regional risk factors.

## Data Availability

The dataset(s) supporting the conclusions of this article were freely publicly available.
